# Supplementary material for: A comprehensive study of metabolite genetics reveals strong pleiotropy and heterogeneity across time and context
Source: Nat Commun. 2019 Oct 21;10:4788. doi: 10.1038/s41467-019-12703-7 (PMC6803661; doi:10.1038/s41467-019-12703-7)
Supplement: Supplementary file 1 — Supplementary Information [file 41467_2019_12703_MOESM1_ESM.pdf]

Supplementary Information

A comprehensive study of metabolite genetics reveals strong pleiotropy and heterogeneity across time and context

Gallois et al.

Contents

Supplementary Methods 2
CMS overview 2
Covariates pre-selection method 2
Definition of region using LD blocks 3
Mapping of gene with top associated variants 3
Comparison and replication with previous GWAS 3
Clustering of genes 4
LIPC fine mapping 4
Supplementary Notes 5
Supplementary Note 1: Association with diseases 5
Supplementary Note 2: Clustering of all genes 5
Supplementary Note 3: Detail results from *LIPC* fine mapping 5
Supplementary Tables 7
Supplementary Table 1: Reference studies used for count of new signals and replication analysis 7
Supplementary Table 2: Principal component analysis of metabolites associated with master regulators 8
Supplementary Table 3: SNPs with highest sum of posterior probabilities of causality across 75 traits 8
Supplementary Table 4: Trend test for the top SNPs of regulator genes 9
Supplementary Table 5: Correlation of fixed effect sizes between time points 9
Supplementary Figures 10
Supplementary Figure 1: Metabolites pairwise correlation 10
Supplementary Figure 2: Variance explained by SNP, confounding factors and covariates 11
Supplementary Figure 3: Distribution of variance explained by covariates 12
Supplementary Figure 4: Effect size in METSIM as a function of -log10(P) in Kettunen et al. 13
Supplementary Figure 5: Distribution of associations per gene 14
Supplementary Figure 6: Distribution of associations per gene 15
Supplementary Figure 7. Expected CMS computation time relative to the number of covariates 16
Supplementary Figure 8. Covariate pre-selection procedure 17
Supplementary Figure 9. Outcome variance explained by pre-selected covariates 18
Supplementary References 19

Supplementary Methods

CMS overview

Consider testing the association between a genotype *G* and a phenotype Y_1. When there exists another measured variable Y_2 correlated with the outcome Y_1 because both variables depend on the same unmeasured risk factor (say *U*), then Y_2 can be considered a proxy for that risk factor *U*. As a proxy, Y_2 can potentially be used as a covariate when regressing Y_1 on *G*. Adjusting Y_1 for Y_2 can substantially reduce the residual variance of Y_1, increasing power to detect *G*-Y_1 association. However, as we discussed in previous work[1], when Y_2 depends on the predictor *G*, using it as a covariate faces a multicollinearity issue, further leading to both false positive and false negative results depending on the underlying causal structure of the data. The core of the *CMS* method is a principled approach to selecting a set of covariates $Y_{k \neq 1}$ that are correlated with the phenotype, but not with the genotype tested, thereby reducing phenotypic variance independent of the genotype and concomitantly increasing power. Our previous work showed that a naïve solution consisting in filtering out covariates based on a *p*-value threshold from the association test between each covariate and the predictor (e.g. testing whether *G*-Y_2 association *p*-value is < 0.05) results in an overall type I error inflation. Instead, we developed a heuristic that uses conditional mean and variance of the parameters in question. In brief, consider $\hat{\delta}$ and $\hat{\beta}$, the marginal estimated regression coefficients between *G* and Y_2, and between *G* and Y_1 (not adjusted for Y_2), respectively, and let $\hat{\gamma}$ be the estimated correlation between Y_1 and Y_2. The central advance of *CMS* is the implementation of an inclusion threshold based on $\mathbb{E}(\hat{\delta}|\hat{\beta})$ and $var(\hat{\delta}|\hat{\beta})$ under a complete null model ($\delta = \beta = 0$).

Covariates pre-selection method

To address the high computational burden of *CMS*, we applied some additional pre-filtering to $Y_{k \neq 1}$ before applying *CMS*. Indeed, *CMS* computation time increases with the number of covariates (N) with a complexity of $O(N^2)$ (**Supplementary Figure 7**). Thus, instead of using about 150 candidate covariates as *CMS* input for each outcome tested, we focused only on the subset explaining the largest (but not too large, *see next section*) amount of the primary outcome variance. We considered two approaches: 1) a naïve one, only based on the marginal correlation between each covariates and the outcome tested, and 2) an alternative one using existing model selection methods -i.e. *Akaike information criterion* (AIC) and *Bayesian information criterion* (BIC)- in order to avoid selecting sets of highly correlated covariates to avoid redundancy. The two strategies are described in **Supplementary Figure 8**. Overall, exploratory analyses in the METSIM data showed that a total number of covariates *N*=30 was enough in most cases to capture the vast majority of the primary outcome variance (**Supplementary Figure 9**). As expected, the naïve approach requires a larger number of covariates than model selection-based approaches to explain the same amount of total variance. There was no observable qualitative difference between AIC and BIC analyses, we therefore only present results from the AIC analyses.

Besides pre-selecting covariates for computational reason, we also applied some additional filtering to maximize robustness. Indeed, the *CMS* principle paper[2] showed a potential increased risk of false positive when including covariates with extremely high correlation with the primary outcome. To address this potential issue, we used an arbitrary absolute correlation threshold of $T = 0.7$ (reflecting the amount of outcome variance explained) above which candidate covariates were automatically filtered out. Furthermore, previous work also showed potential robustness issues when covariates are either parent or linear combination of the outcome tested. To address this issue, we excluded from the set of initial covariates all secondary outcomes that were in the same biological group as the primary outcome. For example, for the GWAS of total lipids in large VLDL (L_VLDL_L), we excluded all other VLDL related variables from the list of candidates' covariates. All exclusions and pre-filtering are listed in **Supplementary Data 1.**

Definition of region using LD blocks

In our analysis, we summarized results per region, using linkage disequilibrium (LD) blocks computed by *Berisa et al*[3]. In brief, they computed blocks using European data from 1000 Genomes phase 1 dataset. The mean block size was set at 10,000 SNPs. They first computed covariance matrix for all pair of SNPs, and then derived a matrix of squared Pearson product moment (where each coefficient is obtained dividing covariance $C_{i,j}$ by the product of $C_{i,i}$ and $C_{j,j}$). They converted this last matrix to a vector by summing antidiagonals, then they applied a low-pass filter to filter out high-frequency fluctuation in the signal. Eventually, they performed local search around minima to define LD blocks. Here we used blocks pre-computed using the 1000 Genomes Europeans individual data as a reference panel for ease of comparison with other studies which include other European ancestry. There were a total of 1703 blocks, with a minimum and maximum length of 10Kb and 26Mb, respectively, and an average size of 1.6 Mb. However, to ensure the map of blocks for Finns and other European population was comparable, were re-computed LD blocks using genotype data from 1000 Genomes Finnish participants. We found only minimal variation with less than 3% of our associations being impacted. In **Supplementary Data 3, 5, 6, 10** and **12**, we summarized results per regions by keeping for each region the SNP with the lowest p-value obtained by either *STD* or *CMS* test.

Mapping of gene with top associated variants

Parsing of the results and assignment to genes was performed through a multi-steps procedure. First, we selected all SNPs-metabolites associations with *STD* or *CMS* *p*-value under the genome-wide significance threshold after correction for multiple testing ($P < 1.28 \times 10^{-9}$). The resulting 3289 SNP-metabolite associations are listed in **Supplementary Data 2**. Second, we used the UCSC database to assign a gene to each SNP. For SNPs, we used table *snp150* with columns *name, chrom, chromStart* and *chromEnd*, corresponding to rs number, chromosome and position. For Genes, we used table *refFlat* with columns *geneName, chrom, txStart* and *txEnd* corresponding to gene name, chromosome and transcription start and end. If the SNP position was in to one or more genes transcription areas, or in an inter-genic area, we kept the closest gene. Third, we grouped these results by regions, keeping the SNP with the minimum *p*-value (either in *STD* or *CMS* approach) in each region-metabolite association. These 588 locus-metabolite associations are listed in **Supplementary data 3**. Finally, to determine which associations were new, we filtered region-metabolite associations with *p*-values above our significance threshold in replication studies. The complete list of 228 new associations is in **Supplementary Data 5**. New results after grouping the 228 region-metabolite associations per gene are presented in **Table 1**. Comparisons with other GWAS, after grouping the 588 region-metabolite associations, are presented in **Supplementary Data 5**. In **Figure 1**, we present the repartition of the 588 associations per metabolite, while corresponding data are provided in **Supplementary Data 4**. In **Figure 3**, we present the 70 genes and the 147 metabolites involved in locus-metabolite associations.

Comparison and replication with previous GWAS

We used nine metabolite GWAS studies for comparison and replication purposes[4-12], described in **Supplementary Table 1**. Five of them considered a range of metabolites[5-9] and had sample size from 2,076 to 24,925, and two focused on a small set of metabolites[4,11], and each included approximately 8,000 individuals. The two remaining studies focused only on total lipids (TC, TG, HDL, and LDL) and had very large sample size of 188,577[10] and 616,626[12]. The Kettunen et al study[8] had the largest number of overlapping metabolites with our study (N=114) while at the same time a large sample size (N=24,925) and therefore contributed the most to

both replication and comparison. Note that the Davis et al study[9] had a strong sample overlap with our analysis and was therefore not used for replication purposes. All these GWAS were performed using standard linear regressions adjusted for known confounding factors such as age, sex, and principal components of the genotypes.

We first used the 7 studies that considered a range of metabolites, plus the Klarin et al study of total lipids[12] to identified previously known region-metabolite association. Out of the 451 region-metabolite pairs were available for comparison across all studies, 360 (80%) showed significant association at the $1.28x10^{-9}$ significance threshold used in our discovery study, and 228 were considered new. Among these new association we further assessed the replication of the best SNP and the same metabolite across studies using the Kettunen et al study[8]. Overall, 68.2% of SNP-metabolite associations had p-value below the nominal threshold of 5% (**Supplementary Data 5**). Finally, we also used genome-wide summary statistics for total lipids from Willer et al[10] to assess previous associations between our top SNPs from the new locus-metabolite associations (N=87, note than the same SNP can exist multiple times in **Supplementary Data 5**). We found that 76 (87%) showed nominal significance with at least one of the four phenotypes.

Clustering of genes

We applied a hierarchical clustering using the *hclust()* R function[13] to identified pattern within the master regulator genes. We used the most common approach: the centroid method and Euclidian distance to quantify cluster dissimilarity, which we applied to the relative number of hits for the lipoprotein type -i.e. the group with the strongest heterogeneity and the less likely to change. The clusters were relatively robust to change in the method (e.g. Ward and median methods generated similar clusters), but we did observe some variability when changing the metric used to derive the distance matrix (e.g. maximum, manhattan), the input data (absolute vs relative number of hits), and the set of features considered (lipoprotein type, size and class).

A visual inspection of the dendrogram suggests 3 primary clusters. We derived the silhouette for various number of clusters using the same dissimilarity matrix as the one used in the hierarchical clustering (i.e. the Euclidian distance) after cutting the dendrogram based on phylogeny heights. We obtained the following average silhouette: 0.608, 0.645, 0.622, 0.598, for 2, 3, 4 and 5 clusters respectively, thus confirming three clusters as the most parsimonious model. Note that for 6 or more clusters the silhouette could not be derived because of the presence of clusters of size 1.

LIPC fine mapping

An individual locus might harbor hundreds of trait-associated variants. To prioritize potentially causal variants, we explored the identified locus using FINEMAP software[14] using the region harboring association with the *LIPC* gene. FINEMAP utilizes shotgun stochastic search algorithm to identify the most likely causal variants within a trait-associated locus. FINEMAP software requires SNP association statistics and their correlation matrix as an input. For *LIPC*, we focus on the sub-region spanned by the SNPs associated with any metabolite and expanding it by 750kb on each side. This resulted in a 1.5Mb region (chr15:57,935,995-59,498,577). We increased the SNP density of this region by imputing new SNPs using minimac and the 1000 Genome phase 1 v3 panel. After imputation our region contained 5,100 SNPs with MAF>0.01. We computed the variant correlation matrix using the METSIM subjects. We applied FINEMAP with default settings for each of the 75 metabolites associated separately. To select the main causal SNPs, we summed the posterior probability for each SNPs across the 75 phenotypes to form a single probability score. We arbitrarily declared the SNPs value of this score

above 10 as best causal candidate SNP. We next explored the functional annotation of theses SNPs. We first search for previous association of those SNPs in the GWAS catalog[15] and *Pubmed* search. We then used *HaploReg v4.1*[16] to check if the potentially causal SNPs were (i) in promoter or enhancer regions (according to the H3K4me1/H3K4me3 and H3K27ac/H3K9ac peaks), (ii) in transcription factors binding sites, (iii) missense variants.

Supplementary Notes

Supplementary Note 1: Association with diseases

We compared our gene-metabolites association results with previous GWAS on coronary heart disease (CHD)[16], body mass index (BMI)[17]and type 2 diabetes (T2D)[18] (**Supplementary Data 6**). We considered here the 70 genes associated with at least one metabolite. We observed substantial enrichment for nominal significance, with 25, 7, and 11 of these genes showing *p*-value below the 5% significance threshold for CHD, BMI and T2D, respectively. Among those, *CELSR2*, *PSRC1* and *LDLR* were genome-wide significant (P = 9.01×10^{-19}, P = 5.20×10^{-17} and P = 1.42×10^{-13} respectively) with CHD, while *CELF1* and *MTCH2* were genome wide significant (P = 2.24×10^{-13} and P = 1.41×10^{-13}) with BMI. Conversely, no association showed genome-wide significance with T2D. To quantify further the observed enrichment for association with these phenotypes, we derived the *q*-values for all SNPs per disease[17]. Given a false discovery rate (FDR) at 10%, we observed 30 significant genes for CHD, 5 for BMI and 4 for T2D (in bold in **Supplementary Data 6**).

Supplementary Note 2: Clustering of all genes

We also applied the clustering approach to all genes associated with at least one lipoprotein. It was not possible here to derive the silhouette because of a one gene cluster (PCDH15) showing up even when considering 2 clusters. Therefore, we defined cluster based on a visual inspection of the dendrogram. The three clusters observed with the master regulators (**Figure 4**) were consistent. The first cluster remain unchanged (*CETP*, *FADS1-2*, *DOCK7* and *LIPC*). One additional gene (*CELF1*) was added to the second one (*TRIB1*, *LPL*, *GCKR*, *GALNT2*, and *APOA5*), and multiple genes (*LINC00663, MLXIPL, FADS3, USP1, MICB, TOMM40*, *CHIC2* and *APOB*) were added to the third one (*PCSK9*, *LDLR, CELSR2* and *APOC1*). Two new clusters appear in this extended analysis: i) the genes *PSRC1, HNF1A* and *MYO1E*, associated with LDL only; ii) *MIR3925, PLTP, MYRF, PTPMT1, PCIF1, APOE, MIR4634, LIPG*, and *LINC02161*, associated only with HDL. The two remaining genes had specific association pattern with PCDH15 being associated with IDL only, and LOC283665 being associated with LDL, HDL and IDL.

Supplementary Note 3: Detail results from *LIPC* fine mapping

We cross-referenced top variants of these three signals identified in the fine mapping of *LIPC* with GWAS of common human diseases[18], and functional annotations from *Haploreg*[16]. The first signal is composed only of SNP rs10468017, which was previously strongly associated with age-related macular degeneration (AMD)[19-21]. It is located in a region harbouring H3K4me1/H3K4me3 and H3K27ac/H3K9ac marks of promoter and enhancer in adipose derived Mesenchymal Stem Cell Cultured Cells. This variant was also reported to be associated with *LIPC*

expression in human liver tissue in a previous study[22], suggesting a potential mode of action through the regulation of *LIPC* expression.

The second signal includes 4 SNPs in complete linkage disequilibrium that were previously associated with hypertension[23], and also AMD[24,25]. It colocalizes with histone marks of promoters and enhancers in liver. These SNPs are also in a region bound by 4 transcription factors: *FOXA1* (rs1077834); *FOXA1* and *FOXA2* (rs1800588); and *RXRA* and *USF1* (rs2070895). Among those transcription factors, *USF1* has been associated with low-density lipoprotein cholesterol levels, triglycerides[26,27], and combined hyperlipidemia[28,29]. Furthermore, *USF1* has been implicated in the expression of hepatic lipase[30], making rs2070895 the strongest candidate for potential functional effects through differential regulation of *LIPC*.

Finally, the last signal included 2 SNPs, among which rs113298164 clearly harboured the highest number of relevant bio-features. It is a rare missense mutation in a region having promoter histone marks in hESC Derived CD184+ Endoderm Cultured Cells. The SNP is also detected by GERP[31] as part of a sequences that is constrained across mammalian genomes. It induces a T405M mutation in *LIPC* protein and is referenced as involved in hepatic lipase deficiency[32].

Supplementary Tables

Supplementary Table 1: Reference studies used for count of new signals and replication analysis

Reference	Title	Sample size	Ancestry	Metabolites measured	Metabolites overlap
Rhee et al	A genome-wide association study of the human metabolome in a community-based cohort	2,076	European	217	15
Shin et al	An atlas of genetic influences on human blood metabolites	7,824	European	486	16
Mozaffarian et al	Genetic loci associated with circulating phospholipid trans fatty acids: a meta-analysis of genome-wide association studies from the CHARGE Consortium	8,013	European	5	2
Kettunen et al	Genome-wide study for circulating metabolites identifies 62 loci and reveals novel systemic effects of*LPA*	24,925	European	123	114
Rhee et al	An exome array study of the plasma metabolome	3,604	European	217	16
Davis et al	Common, low-frequency, and rare genetic variants associated with lipoprotein subclasses and triglyceride measures in Finnish men from the METSIM study	8,372	European	72	72
Willer et al	Discovery and refinement of loci associated with lipid levels	188,577	European	4	4
Klarin et al	Genetics of blood lipids among ~300,000 multiethnic participants of the Million Veteran Program	616,626	mostly European	4	4
Teslovich et al	Identification of seven novel loci associated with amino acid levels using single-variant and gene-based tests in 8545 Finnish men from the METSIM study	8,545	European	9	9

Supplementary Table 2: Principal component analysis of metabolites associated with master regulators

	Including all metabolites					Including lipoprotein only				
	Total number of association	Number of PC required to explained X% the total variance				Total number of association	Number of PC required to explained X% the total variance			
		X=50%	*X=90%*	*X=99%*	*X=99.9%*		*X=50%*	*X=90%*	*X=99%*	*X=99.9%*
TRIB1	23	1	2	6	11	18	1	2	4	8
LPL	29	1	3	9	16	19	1	3	6	9
GALNT2	28	1	3	8	12	23	1	2	5	9
GCKR	38	1	4	11	17	27	1	2	5	10
APOA5	53	1	4	15	25	36	1	3	9	16
PCSK9	45	1	4	10	19	37	1	4	9	16
LIPC	75	2	7	19	36	46	2	5	12	22
LDLR	39	1	3	8	16	31	1	2	7	13
CELSR2	23	1	2	5	11	18	1	1	4	9
APOC1	33	1	2	7	15	27	1	2	6	12
CETP	51	2	5	12	23	41	2	5	11	19
FADS1-2	19	2	5	11	15	9	1	3	5	6
DOCK7	20	1	4	9	14	10	1	3	5	8

Supplementary Table 3: SNPs with highest sum of posterior probabilities of causality across 75 traits

SNP	Chr.	Position	MAF	Distance to LIPC	Sum of post prob	linkage disequilibrium (r^2) in METSIM data: rs10468017	rs1077835	rs1077834	rs1800588	rs2070895	rs113298164	rs111285504
rs10468017	15	58,678,512	0.328	45,662	32.2	**1.**	0.003	0.003	0.003	0.003	7.6E-4	7.6E-4
rs1077835	15	58,723,426	0.281	748	10.7	0.003	**1.**	**1.**	**1.**	**1.**	0.003	0.003
rs1077834	15	58,723,479	0.281	695	10.7	0.003	**1.**	**1.**	**1.**	**1.**	0.003	0.003
rs1800588	15	58,723,675	0.281	499	10.7	0.003	**1.**	**1.**	**1.**	**1.**	0.003	0.003
rs2070895	15	58,723,939	0.281	235	10.7	0.003	**1.**	**1.**	**1.**	**1.**	0.003	0.003
rs113298164	15	58,855,748	0.015	0	12.3	7.6E-4	0.003	0.003	0.003	0.003	**1.**	**1.**
rs111285504	15	58,859,395	0.015	0	12.3	7.6E-4	0.003	0.003	0.003	0.003	**1.**	**1.**

Supplementary Table 4: Trend test for the top SNPs of regulator genes

	Statin interaction			Delta		
	sumZ coef.	*SD*	*pval*	*sumZ coef.*	*SD*	*pval*
APOA5	6.6	567.4	0.78	-109.1	567.4	**4.6E-6**
APOC1	-89.0	315.5	**5.3E-7**	-54.8	315.5	**2.0E-3**
CELSR2	-15.4	210.0	0.29	-33.0	210.0	**0.023**
CETP	-61.3	759.8	0.026	-52.5	759.8	**0.057**
DOCK7	1.7	63.1	0.83	-24.6	63.1	**1.9E-3**
FADS2	0.1	49.6	0.98	-18.3	49.6	**9.4E-3**
GALNT2	-14.7	245.8	0.35	-38.5	245.8	**0.014**
GCKR	7.7	339.1	0.68	-62.6	339.1	**6.7E-4**
LDLR	-68.9	352.4	**2.4E-4**	-78.4	352.4	**3.0E-5**
LIPC	4.1	1128.2	0.90	-49.7	1128.2	0.14
LPL	11.5	231.8	0.45	-10.4	231.8	0.50
PCSK9	34.0	530.3	0.14	2.7	530.3	0.91
TRIB1	-44.1	134.0	**1.4E-4**	-38.9	134.0	**7.7E-4**

Supplementary Table 5: Correlation of fixed effect sizes between time points

Parameter	Correlation
Intercept	-0.696
age	0.641
age2	0.626
PC1	0.822
PC2	0.903
PC3	0.635
PC4	0.609
PC5	0.337
PC6	0.333
PC7	0.290
PC8	0.506
PC9	0.826
PC10	0.114

Supplementary Figures

Supplementary Figure 1: Metabolites pairwise correlation

Illustration of the 158 analyzed metabolites pairwise correlation. Negative values are in blue and positive values are in red. The inner histogram shows the distribution of the pairwise correlation across all metabolites pairs.

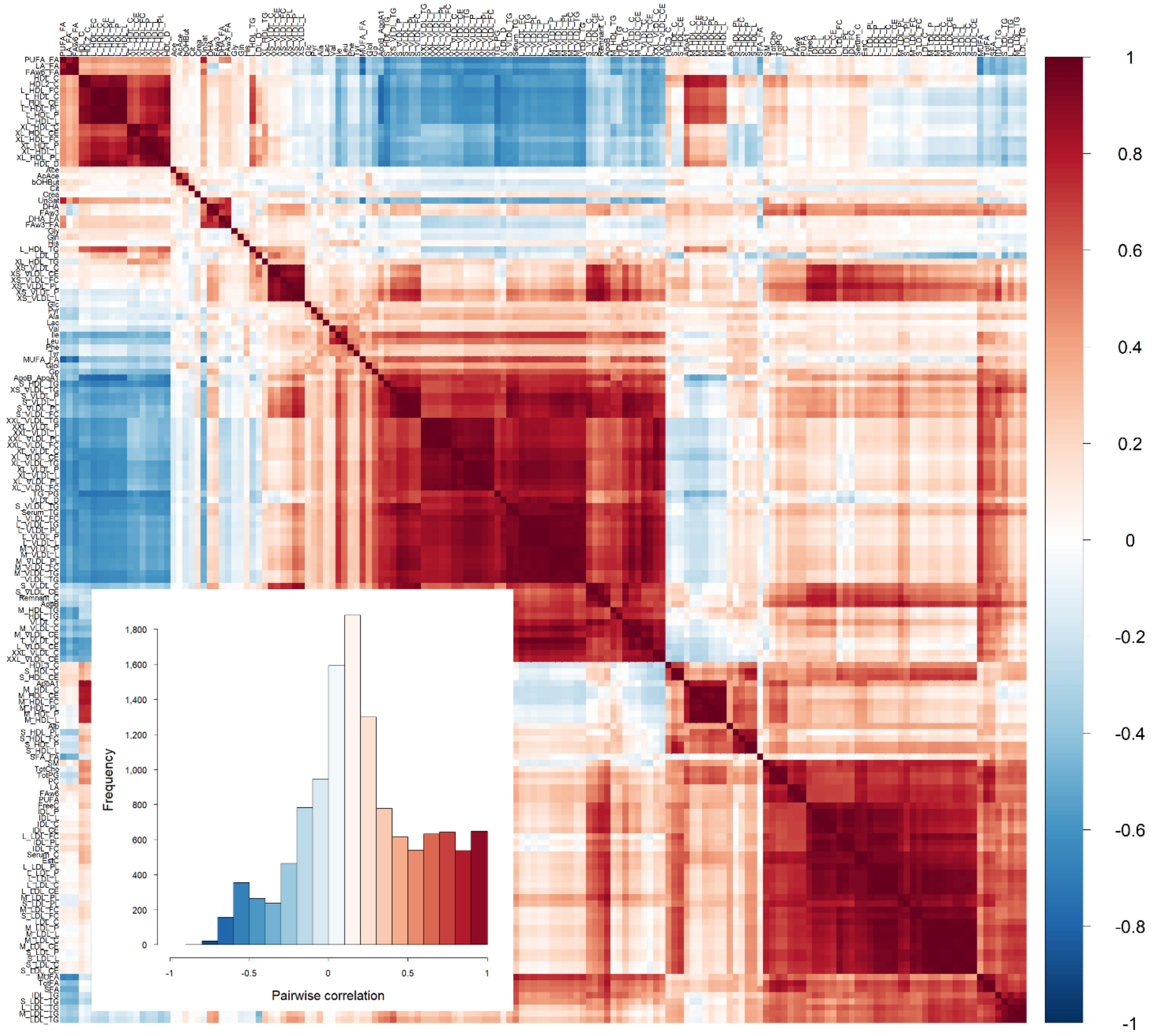

Supplementary Figure 2: Variance explained by SNP, confounding factors and covariates

Example of gain in power achieved by *CMS* using the reported association between leucine and rs1260326. Each panel represents leucine variance. (**a**) In standard model, SNP and confounding factors explain a small fraction of leucine variance. Residual variance equals 96%. (**b**) Adding preselected covariates in the model without filtering for collinearity with the SNP tested. Residual variance equals 9%. (**c**) After exclusion of covariates likely associated with the SNP by *CMS*. (**d**) Final model with CMS-selected covariates. Residual variance equals 34 %.

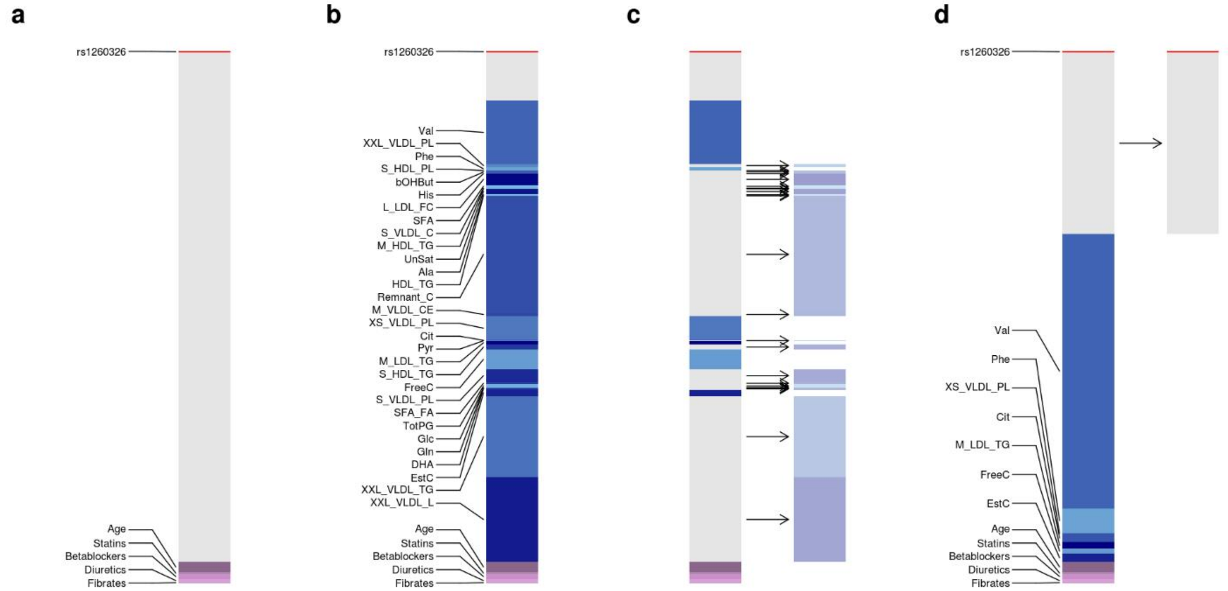

Supplementary Figure 3: Distribution of variance explained by covariates

The histogram shows the outcome variance explained by covariates selected by *CMS* across the 158 metabolites by 600,000 SNPs analyzed (barplot and Y axis on the left). For the lower bound of each bar category, we derived the equivalent sample size that is achieved (red curve, and red Y axis on the right).

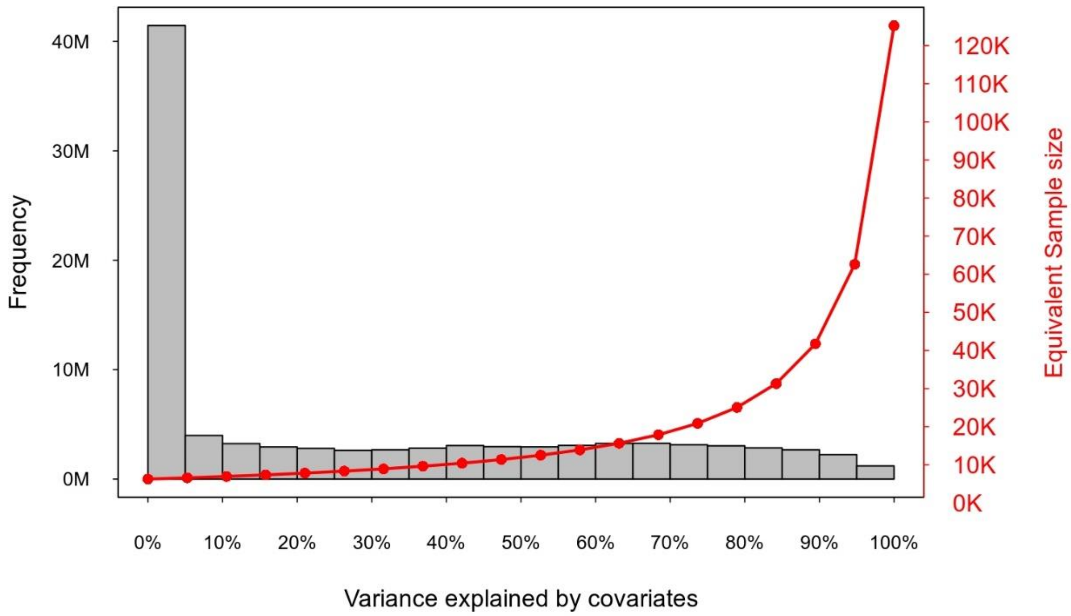

Supplementary Figure 4: Effect size in METSIM as a function of -log10(P) in Kettunen et al.

To explore the performances of the replication stage, we plotted the 442 region-metabolite association with data for both the discovery stage in METSIM and replication stage in Kettunen et al 2016. The Y axis shows the variance explained by the top variant (defined as the squared correlation) in METSIM, and the X axis shows the -log10(P-value) observed in the Kettunen et al. study for the same variant. The size of the points are proportional to the gain in power achieved by *CMS*. The green and blue dashed lines show the significance threshold at 5e-9 and 0.05, respectively. For clarity we cut the Y and X axis at 0.02 and 100, removing a few data points outside that range. The strong correlation indicates ((ρ=0.63) that lack of replication was mostly due to limited power in the replication for SNPs discovered thanks top the boost in power by CMS.

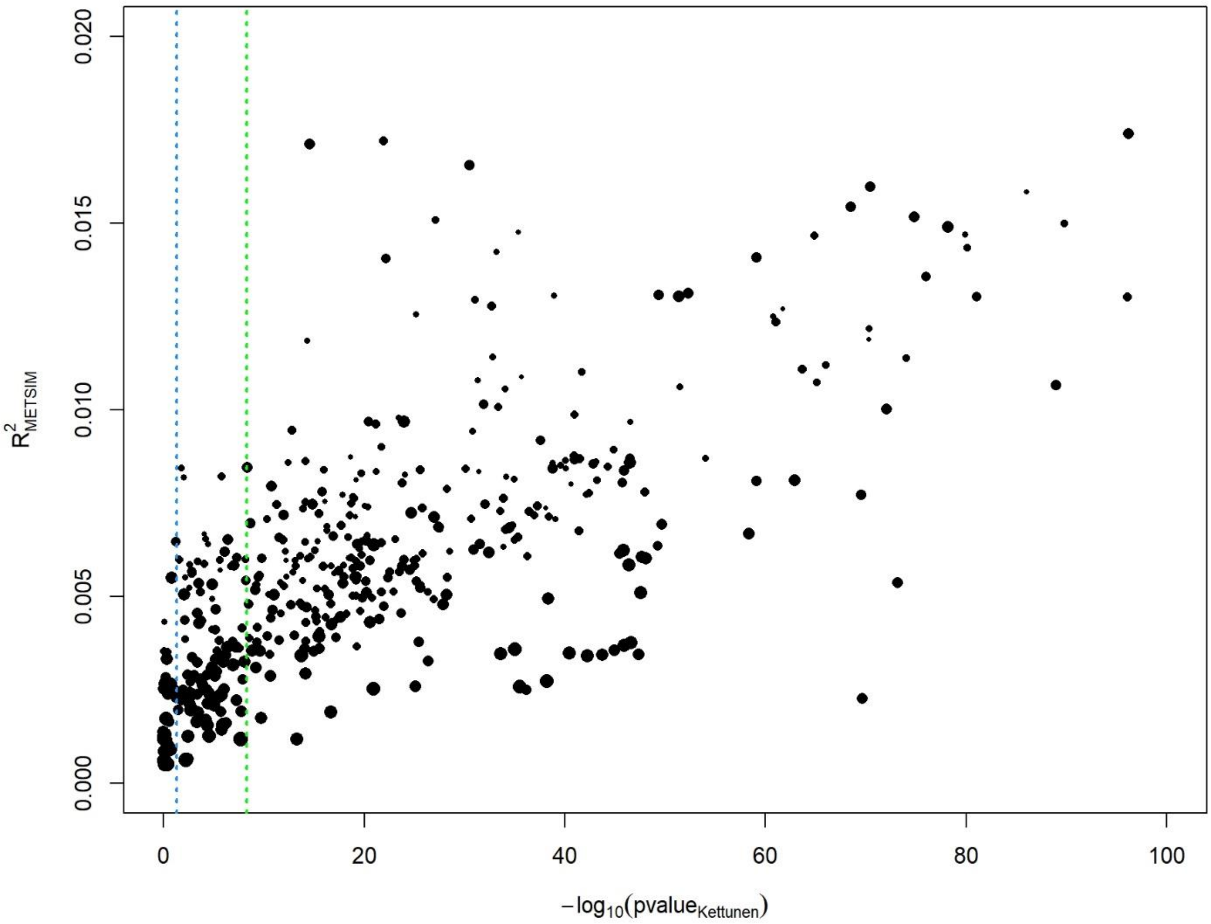

Supplementary Figure 5: Distribution of associations per gene

Barplots represent the number of metabolites associated for each of the 70 genes reported in Supplementary Data 4. Contribution of the seven metabolite groups is showed by different colors. Genes are ordered by their respective total number of associations. The red line shows the cumulated percentage of the total number of gene-metabolite association reported in our study (right axis).

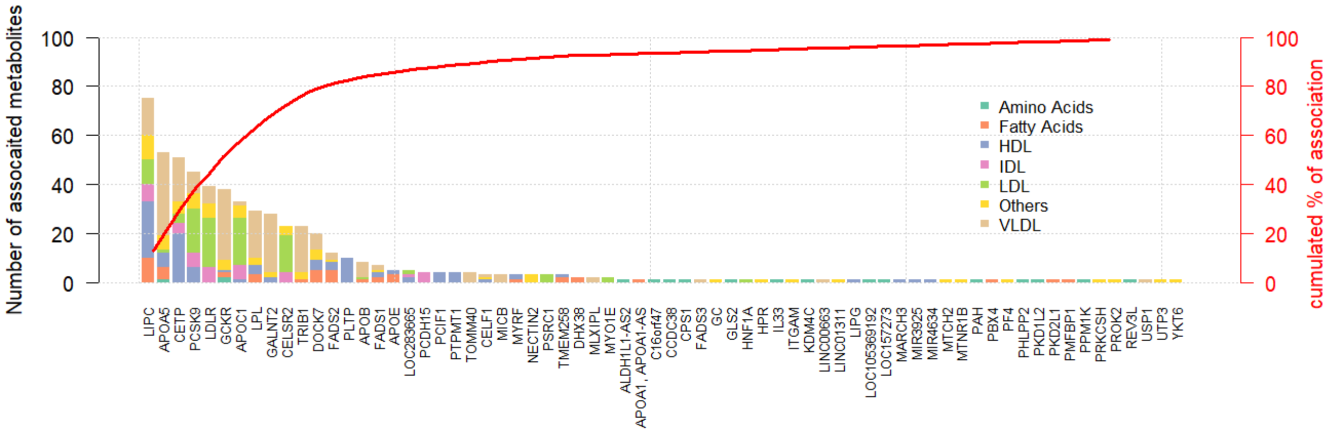

Supplementary Figure 6: Distribution of associations per gene

We performed a hierarchical clustering of the association between the between all genes associated with at least one lipoprotein and the lipoprotein type (a). Further panels show the total number of associations, the number of associations with lipoprotein, and the total number of top associated SNP (b); the count of association hits by lipoprotein type (c), their size (d), and class (e). The background colours represent the relative proportion of association within each gene-item stratum, highlighting heterogeneity in the distribution of signal.

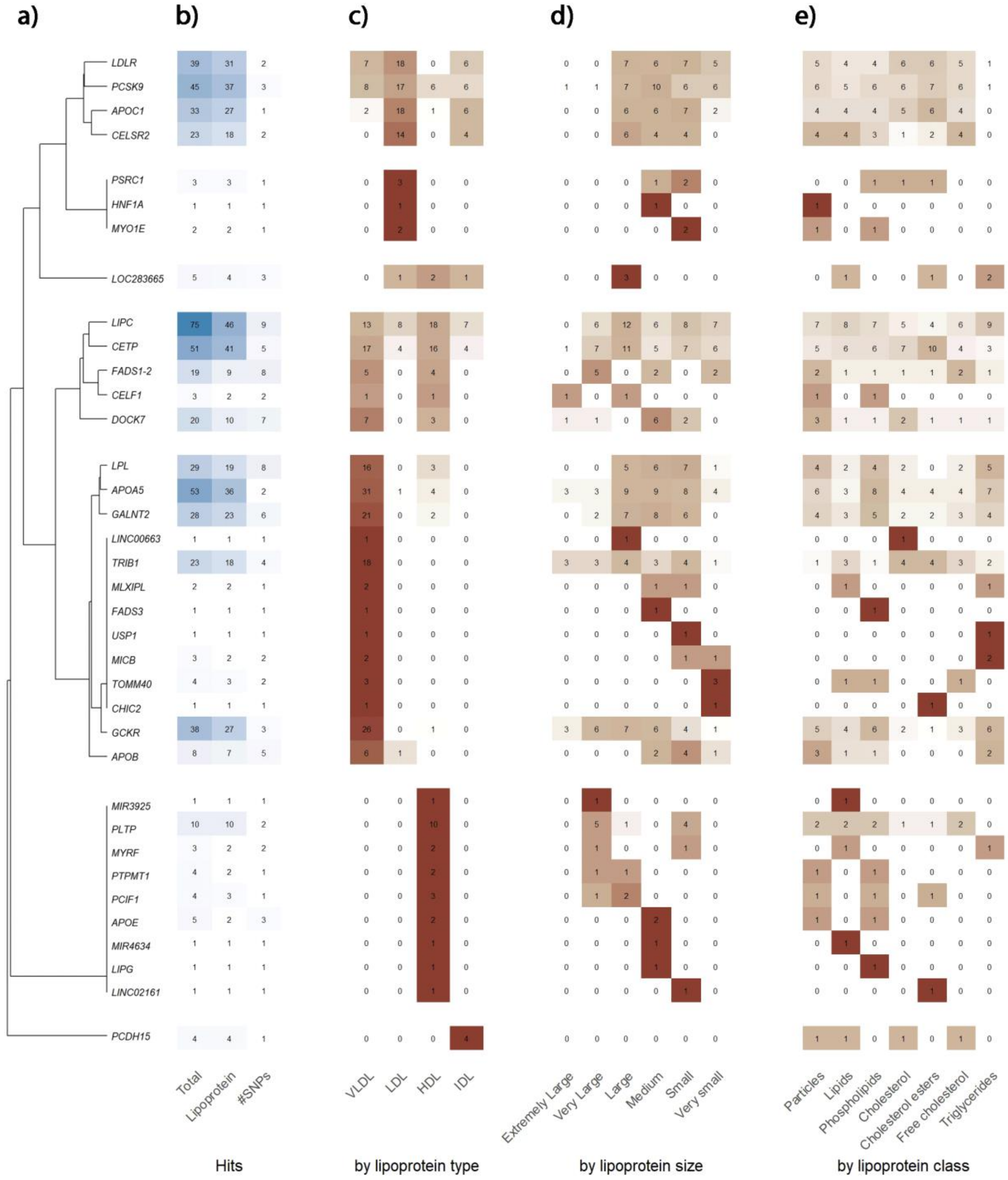

Supplementary Figure 7. Expected CMS computation time relative to the number of covariates

Simulation of 500 phenotypes with a normal distribution. We run CMS on the first phenotype – the outcome – varying the number of covariates from 1 to 200. We measure computation time of CMS on each model. This plot shows that complexity is in $O(N^2)$ when N is the number of covariates.

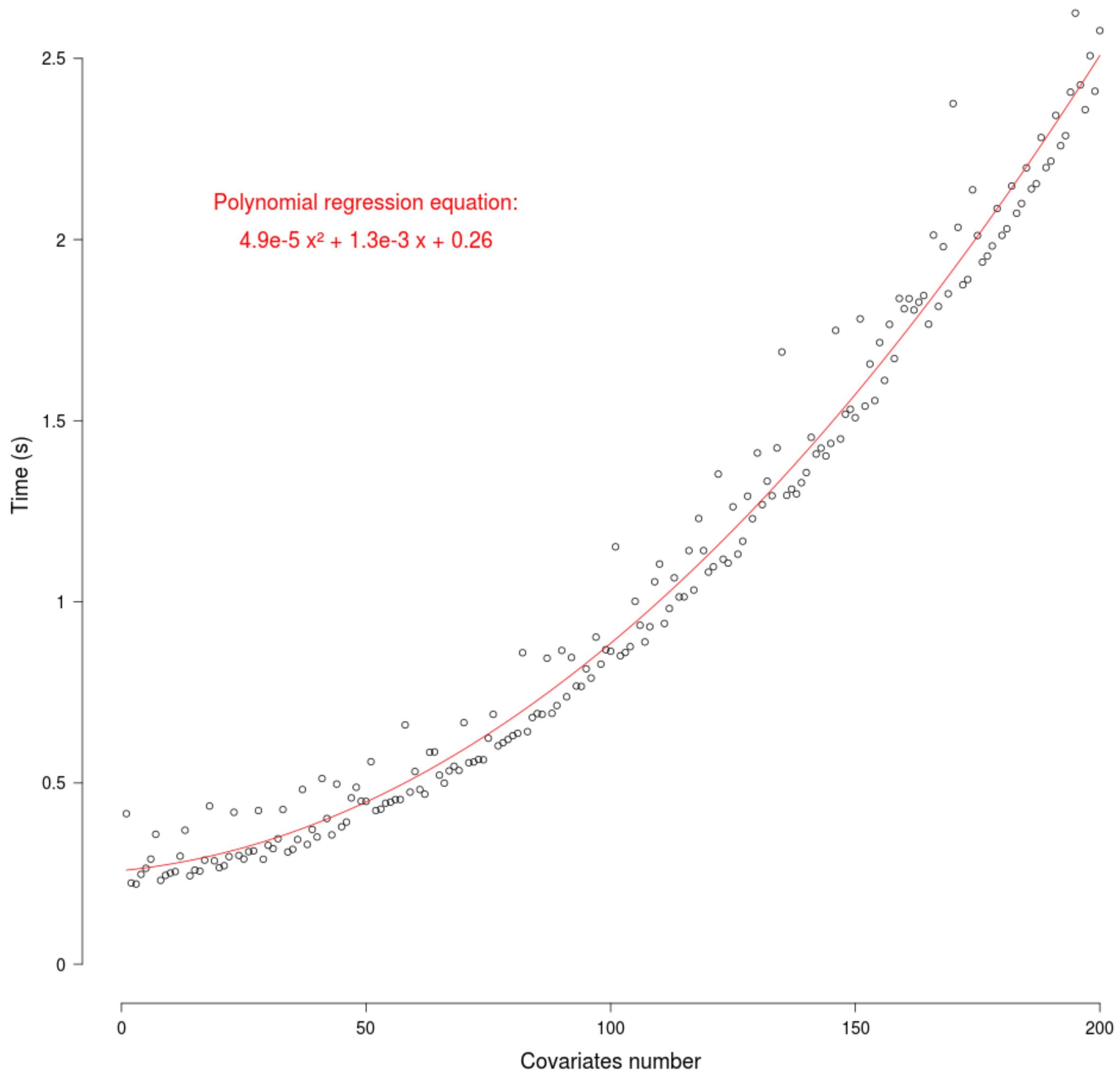

Supplementary Figure 8. Covariate pre-selection procedure

Presentation of the two methods tested to pre-select covariates before applying CMS. (**a**) Naïve approach where we compute outcome variance explained by each variable. We sort the variables according to this criterion and select the N first as covariates. (**b**) Approach using Akaike Information Criteria (AIC). We first add the variable that explains the highest outcome variance. Then we compute AIC for each possible model and add iteratively covariates, stopping when we have N of them.

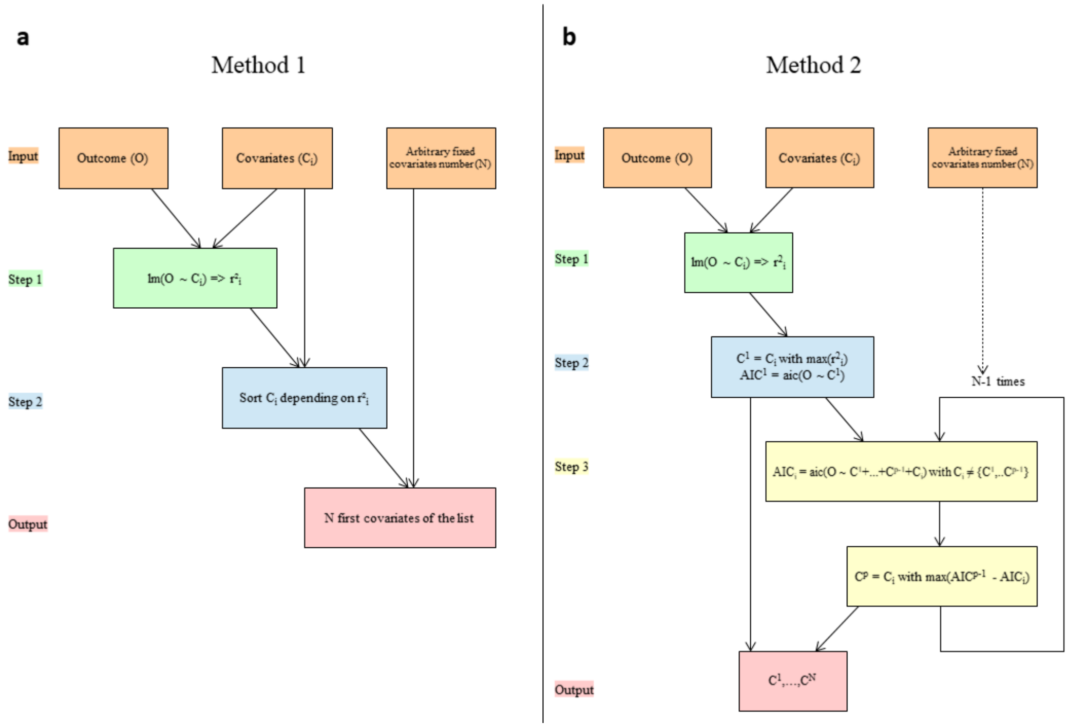

Supplementary Figure 9. Outcome variance explained by pre-selected covariates

We applied the procedure described in Supplementary figure 5 in METSIM data, in Leucine. Plots show outcome explained variance depending on the number of covariates incorporated in the model, for method 1 (in red) or 2 (in blue). We applied the thresholds 0.1 (a), 0.3 (b), 0.5 (c), 0.7 (d), 0.9 (e), 1 (f) on outcome variance explained by each covariate added in the model. Dashed grey line shows that most of the outcome variance can be explained by approximately 30 covariates.

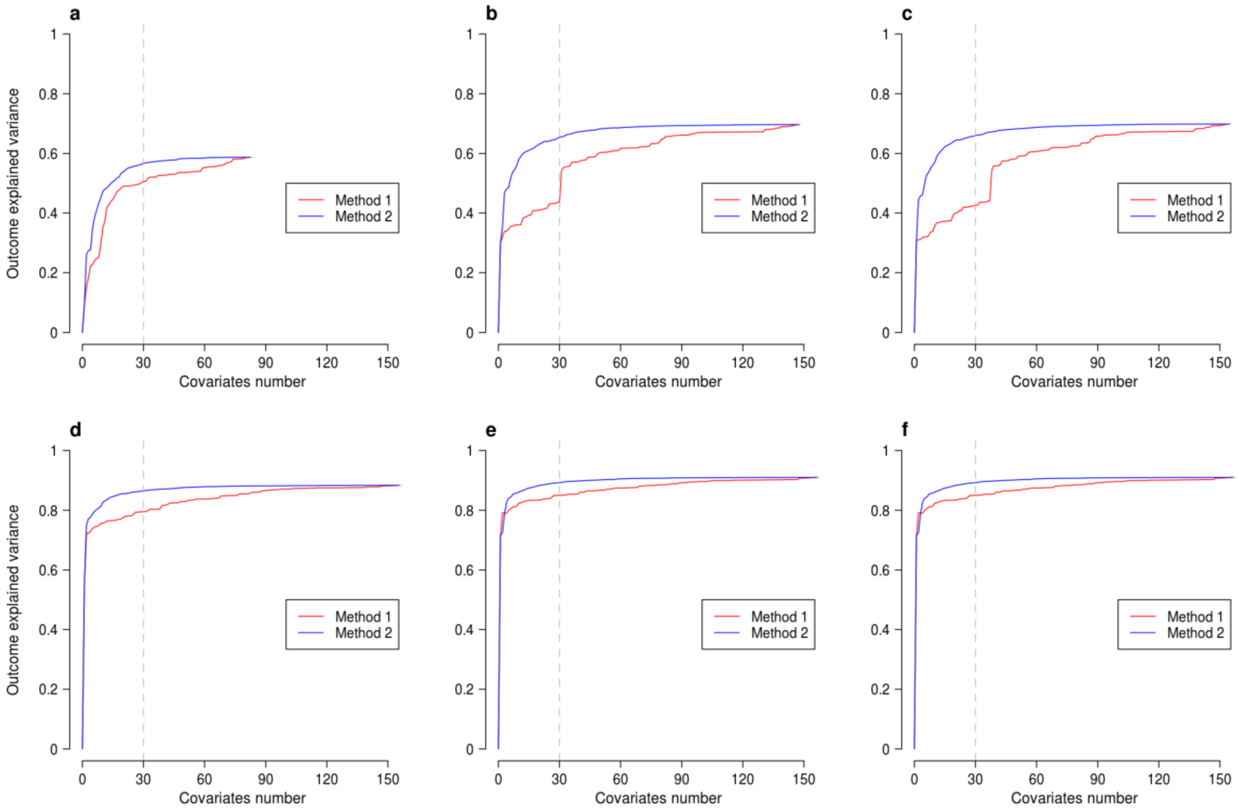

Supplementary References

1. Aschard, H., Vilhjalmsson, B.J., Joshi, A.D., Price, A.L. & Kraft, P. Adjusting for heritable covariates can bias effect estimates in genome-wide association studies. *Am J Hum Genet* **96**, 329-39 (2015).
2. Aschard, H. *et al.* Covariate selection for association screening in multiphenotype genetic studies. *Nat Genet* **49**, 1789-1795 (2017).
3. Berisa, T. & Pickrell, J.K. Approximately independent linkage disequilibrium blocks in human populations. *Bioinformatics* **32**, 283-5 (2016).
4. Mozaffarian, D. *et al.* Genetic loci associated with circulating phospholipid trans fatty acids: a meta-analysis of genome-wide association studies from the CHARGE Consortium. *Am J Clin Nutr* **101**, 398-406 (2015).
5. Rhee, E.P. *et al.* An exome array study of the plasma metabolome. *Nat Commun* **7**, 12360 (2016).
6. Rhee, E.P. *et al.* A genome-wide association study of the human metabolome in a community-based cohort. *Cell Metab* **18**, 130-43 (2013).
7. Shin, S.Y. *et al.* An atlas of genetic influences on human blood metabolites. *Nat Genet* **46**, 543-550 (2014).
8. Kettunen, J. *et al.* Genome-wide study for circulating metabolites identifies 62 loci and reveals novel systemic effects of LPA. *Nat Commun* **7**, 11122 (2016).
9. Davis, J.P. *et al.* Common, low-frequency, and rare genetic variants associated with lipoprotein subclasses and triglyceride measures in Finnish men from the METSIM study. *PLoS Genet* **13**, e1007079 (2017).
10. Willer, C.J. *et al.* Discovery and refinement of loci associated with lipid levels. *Nat Genet* **45**, 1274-1283 (2013).
11. Teslovich, T.M. *et al.* Identification of seven novel loci associated with amino acid levels using single-variant and gene-based tests in 8545 Finnish men from the METSIM study. *Hum Mol Genet* **27**, 1664-1674 (2018).
12. Klarin, D. *et al.* Genetics of blood lipids among ~300,000 multi-ethnic participants of the Million Veteran Program. *Nat Genet* **50**, 1514-1523 (2018).
13. Becker, R.A., Chambers, J.M. & Wilks, A.R. *The new S language: a programming environment for data analysis and graphics*, 702 (Wadsworth and Brooks/Cole Advanced Books \\& Software, 1988).
14. Benner, C. *et al.* FINEMAP: efficient variable selection using summary data from genome-wide association studies. *Bioinformatics* **32**, 1493-501 (2016).
15. Welter, D. *et al.* The NHGRI GWAS Catalog, a curated resource of SNP-trait associations. *Nucleic Acids Res* **42**, D1001-6 (2014).
16. Ward, L.D. & Kellis, M. HaploReg: a resource for exploring chromatin states, conservation, and regulatory motif alterations within sets of genetically linked variants. *Nucleic Acids Res* **40**, D930-4 (2012).
17. Storey, J.D. & Tibshirani, R. Statistical significance for genomewide studies. *Proc Natl Acad Sci U S A* **100**, 9440-5 (2003).
18. MacArthur, J. *et al.* The new NHGRI-EBI Catalog of published genome-wide association studies (GWAS Catalog). *Nucleic Acids Res* **45**, D896-D901 (2017).
19. Neale, B.M. *et al.* Genome-wide association study of advanced age-related macular degeneration identifies a role of the hepatic lipase gene (LIPC). *Proc Natl Acad Sci U S A* **107**, 7395-400 (2010).
20. Yu, Y. *et al.* Common variants near FRK/COL10A1 and VEGFA are associated with advanced age-related macular degeneration. *Hum Mol Genet* **20**, 3699-709 (2011).
21. Wang, Y.F. *et al.* CETP/LPL/LIPC gene polymorphisms and susceptibility to age-related macular degeneration. *Sci Rep* **5**, 15711 (2015).

22. Kathiresan, S. *et al.* Common variants at 30 loci contribute to polygenic dyslipidemia. *Nat Genet* **41**, 56-65 (2009).
23. Zhao, X., Ren, Y., Li, H. & Wu, Y. Association of LIPC -250G/A and -514C/T polymorphisms and hypertension: a systematic review and meta-analysis. *Lipids Health Dis* **17**, 238 (2018).
24. Fritsche, L.G. *et al.* A large genome-wide association study of age-related macular degeneration highlights contributions of rare and common variants. *Nat Genet* **48**, 134-43 (2016).
25. Colijn, J.M. *et al.* Increased High-Density Lipoprotein Levels Associated with Age-Related Macular Degeneration: Evidence from the EYE-RISK and European Eye Epidemiology Consortia. *Ophthalmology* **126**, 393-406 (2019).
26. Holzapfel, C. *et al.* Genetic variants in the USF1 gene are associated with low-density lipoprotein cholesterol levels and incident type 2 diabetes mellitus in women: results from the MONICA/KORA Augsburg case-cohort study, 1984-2002. *Eur J Endocrinol* **159**, 407-16 (2008).
27. Coon, H. *et al.* Upstream stimulatory factor 1 associated with familial combined hyperlipidemia, LDL cholesterol, and triglycerides. *Hum Genet* **117**, 444-51 (2005).
28. Di Taranto, M.D. *et al.* Association of USF1 and APOA5 polymorphisms with familial combined hyperlipidemia in an Italian population. *Mol Cell Probes* **29**, 19-24 (2015).
29. Lee, J.C., Lusis, A.J. & Pajukanta, P. Familial combined hyperlipidemia: upstream transcription factor 1 and beyond. *Curr Opin Lipidol* **17**, 101-9 (2006).
30. van Deursen, D. *et al.* Activation of hepatic lipase expression by oleic acid: possible involvement of USF1. *Nutrients* **1**, 133-47 (2009).
31. Davydov, E.V. *et al.* Identifying a high fraction of the human genome to be under selective constraint using GERP++. *PLoS Comput Biol* **6**, e1001025 (2010).
32. Ruel, I.L. *et al.* Characterization of a novel mutation causing hepatic lipase deficiency among French Canadians. *J Lipid Res* **44**, 1508-14 (2003).
