# Supplementary material for: A comprehensive study of metabolite genetics reveals strong pleiotropy and heterogeneity across time and context
Source: Nat Commun. 2019 Oct 21;10:4788. doi: 10.1038/s41467-019-12703-7 (PMC6803661; doi:10.1038/s41467-019-12703-7)
Supplement: Supplementary file 16 — Description of Additional Supplementary Files [file 41467_2019_12703_MOESM16_ESM.pdf]

Title: Supplementary Data 1:
Description: 158 metabolite measurements

Title: Supplementary Data 2:
Description: Significant SNP- metabolite associations

Title: Supplementary Data 3:
Description: 588 region-metabolite associations resulting from large scale analysis of 158 metabolites in METSIM cohort

Title: Supplementary Data 4:
Description: Repartition of locus - metabolite associations

Title: Supplementary Data 5:
Description: 228 new region-metabolite associations (P < 1.28 x 10-9in reference GWAS metabolites)

Title: Supplementary Data 6:
Description: SNPs from our 588 associations with corresponding p-values in GWAS of CHD, BMI and Type 2 diabetes

Title: Supplementary Data 7:
Description: Alternative SNP-effective Gene mapping for master regulators

Title: Supplementary Data 8:
Description: Posterior probability of being causal in LIPC region

Title: Supplementary Data 9:
Description: Heterogeneity in posterior probabilities of causality across the 75 traits

Title: Supplementary Data 10:
Description: Statin interaction on region-metabolite associations

Title: Supplementary Data 11:
Description: 30 significant SNP associations resulting from large-scale analysis of 158 metabolite differences between baseline and follow-up data in METSIM cohort (P < 5 x 10-8)

Title: Supplementary Data 12:
Description: 8 significant locus associations resulting from large-scale analysis of 158 metabolites differences between baseline and follow-up data in METSIM cohort

Title: Supplementary Data 13:
Description: Heritability computed in individuals present in both baseline and follow-up studies
