# Supplementary material for: A comprehensive study of metabolite genetics reveals strong pleiotropy and heterogeneity across time and context
Source: Nat Commun. 2019 Oct 21;10:4788. doi: 10.1038/s41467-019-12703-7 (PMC6803661; doi:10.1038/s41467-019-12703-7)
Supplement: Supplementary file 17 — Reporting Summary [file 41467_2019_12703_MOESM17_ESM.pdf]

nature research

Corresponding author(s): Hugues Aschard

Last updated by author(s): Dec 10, 2018

Reporting Summary

Nature Research wishes to improve the reproducibility of the work that we publish. This form provides structure for consistency and transparency in reporting. For further information on Nature Research policies, see Authors & Referees and the Editorial Policy Checklist.

Statistics

For all statistical analyses, confirm that the following items are present in the figure legend, table legend, main text, or Methods section.

n/a	Confirmed	
☐	☒	The exact sample size (*n*) for each experimental group/condition, given as a discrete number and unit of measurement
☐	☒	A statement on whether measurements were taken from distinct samples or whether the same sample was measured repeatedly
☐	☒	The statistical test(s) used AND whether they are one- or two-sided<br>*Only common tests should be described solely by name; describe more complex techniques in the Methods section.*
☐	☒	A description of all covariates tested
☐	☒	A description of any assumptions or corrections, such as tests of normality and adjustment for multiple comparisons
☐	☒	A full description of the statistical parameters including central tendency (e.g. means) or other basic estimates (e.g. regression coefficient) AND variation (e.g. standard deviation) or associated estimates of uncertainty (e.g. confidence intervals)
☐	☒	For null hypothesis testing, the test statistic (e.g. *F*, *t*, *r*) with confidence intervals, effect sizes, degrees of freedom and *P* value noted<br>*Give P values as exact values whenever suitable.*
☒	☐	For Bayesian analysis, information on the choice of priors and Markov chain Monte Carlo settings
☒	☐	For hierarchical and complex designs, identification of the appropriate level for tests and full reporting of outcomes
☐	☒	Estimates of effect sizes (e.g. Cohen's *d*, Pearson's *r*), indicating how they were calculated

Our web collection on statistics for biologists contains articles on many of the points above.

Software and code

Policy information about availability of computer code

Data collection	This study did not generated any new data. It uses existing anonymized data from the METSIM study which has been previously described (PMID:28119442)
Data analysis	All analyses were performed using open source code/software<br>- The GWAS association screening was performed using the CMS approach (PMID: 29038595). Code is available at: https://gitlab.pasteur.fr/statistical-genetics/runCMS/<br>- The bivariate heritability analysis was performed using GCTA's bivariate REML (PMID: 22843982)<br>- plotting of the gene-metabolites network was done using Cytoscape (PMID: 14597658)<br>- all others analyses and plots were done using the R software (https://www.r-project.org/)

For manuscripts utilizing custom algorithms or software that are central to the research but not yet described in published literature, software must be made available to editors/reviewers. We strongly encourage code deposition in a community repository (e.g. GitHub). See the Nature Research guidelines for submitting code & software for further information.

Data

Policy information about availability of data

All manuscripts must include a data availability statement. This statement should provide the following information, where applicable:

- Accession codes, unique identifiers, or web links for publicly available datasets
- A list of figures that have associated raw data
- A description of any restrictions on data availability

Genotype and phenotype data from the METSIM cohort are available through METSIM data access committee (http://www.nationalbiobanks.fi/index.php/studies2/10-metsim).

All figures (1 to 7) show results derived using the raw MESTIM data

Complete summary statistics from the GWAS are available at http://statgen.pasteur.fr/Download.html. They have also been posted on the NHGRI-EBI Catalog of published genome-wide association studies, and will become available after the publication of the manuscript.

Field-specific reporting

Please select the one below that is the best fit for your research. If you are not sure, read the appropriate sections before making your selection.

☒ Life sciences ☐ Behavioural & social sciences ☐ Ecological, evolutionary & environmental sciences

For a reference copy of the document with all sections, see nature.com/documents/nr-reporting-summary-flat.pdf

Life sciences study design

All studies must disclose on these points even when the disclosure is negative.

Sample size	Our study included all unrelated individuals from the Finnish Metabolic Syndrome In Men (METSIM) with genome-wide genetic data and metabolites data (N=6,263).
Data exclusions	related individuals were excluded as they could not be analyzed using the chosen methodology.
Replication	We performed in-silico replication, comparing our association results with GWAS summary statistics from previous screening (PMID: 27005778, 24816252, 20686565, and 23823483)
Randomization	NA
Blinding	NA

Reporting for specific materials, systems and methods

We require information from authors about some types of materials, experimental systems and methods used in many studies. Here, indicate whether each material, system or method listed is relevant to your study. If you are not sure if a list item applies to your research, read the appropriate section before selecting a response.

Materials & experimental systems

n/a	Involved in the study	
☒	☐	Antibodies
☒	☐	Eukaryotic cell lines
☒	☐	Palaeontology
☒	☐	Animals and other organisms
☐	☒	Human research participants
☒	☐	Clinical data

Methods

n/a	Involved in the study	
☒	☐	ChIP-seq
☒	☐	Flow cytometry
☒	☐	MRI-based neuroimaging

Human research participants

Policy information about studies involving human research participants

Population characteristics	The METSIM cohort is composed of 10,197 Finnish men from 45 to 73 years old and aimed at investigating non-genetic and genetic factors associated with Type 2 Diabetes and cardiovascular diseases. For each sample, 228 serum metabolites (lipids, lipoproteins, amino acids, fatty acids and other low molecular weight metabolites) measurements were made with nuclear magnetic resonance (NMR) at baseline. A follow-up study was conducted about 5 years after the baseline study. 6,496 participants (64 %) were reexamined with the same protocol and metabolites were measured a second time using the same technology. In our study, we considered 158 variables, including 150 raw measurements and 8 ratios. Besides metabolic measurements, several variables were also available including drug treatment and large group of other phenotypes. All samples were genotyped for 665,478 SNPs using the Illumina OmniExpress chip.
Recruitment	Participants were recruited and examined between 2005 and 2010 in Kuopio town in Eastern Finland.
Ethics oversight	The study was approved by the ethics committee of the University of Kuopio and Kuopio University Hospital, in accordance with the Helsinki Declaration.

Note that full information on the approval of the study protocol must also be provided in the manuscript.
